# Burden of eosinophilic granulomatosis with polyangiitis in Europe

**DOI:** 10.1183/23120541.00912-2023

**Published:** 2024-08-05

**Authors:** Rupert W. Jakes, Namhee Kwon, Lynn Huynh, Jeremiah Hwee, Lee Baylis, Rafael Alfonso-Cristancho, Shawn Du, Anamika Khanal, Mei Sheng Duh, Benjamin Terrier

**Affiliations:** 1 Epidemiology, GSK, London, UK; 2 Clinical Sciences, Respiratory, GSK, London, UK; 3 Analysis Group, Inc., Boston, MA, USA; 4 Epidemiology, GSK, Mississauga, ON, Canada; 5 Global Medical Affairs, GSK, Durham, NC, USA; 6 Value Evidence & Outcomes, GSK, Collegeville, PA, USA; 7 Service de Médecine Interne, Hôpital Cochin, Paris, France

## Abstract

**Background and aims:**

Real-world evidence characterising the burden of eosinophilic granulomatosis with polyangiitis (EGPA) in Europe is limited. The aim of this study was to characterise patients in a large European EGPA cohort.

**Methods:**

This retrospective, non-interventional, longitudinal study (GSK ID: 214661) recruited cross-specialty physicians from France, Germany, Italy, Spain and the UK to conduct medical chart reviews for patients with a physician-confirmed diagnosis of EGPA. Patients were ≥12 years of age at diagnosis with ≥1 year of follow-up data from the first clinical visit with the physician (index date). Outcome measures collected from index date to end of follow-up included clinical manifestations and healthcare resource utilisation (HCRU).

**Results:**

In total, 407 patient medical charts were reviewed by 204 physicians; median (interquartile range) duration of follow-up from index date was 2.2 (1.7−3.5) years. Most patients (73.5%) had asthma. Patients underwent multiple diagnostic assessments, and 74.9% received ≥3 different therapies between diagnosis and end of follow-up (98.8% oral corticosteroids, 63.9% immunosuppressive therapies, 45.5% biologics). During follow-up, 84.5% of patients experienced EGPA clinical manifestations; most were considered moderate or severe and commonly affected the lungs (55.8%; including lung infiltrates 25.8% and severe asthma 24.8%), ear, nose and throat (53.3%), and skin (41.8%). HCRU was substantial: 26.0% of patients made emergency department visits, 36.6% were hospitalised and 84.8% had outpatient visits.

**Conclusions:**

These real-world data show that EGPA presents a substantial burden to patients and the healthcare system. Earlier and better differential diagnosis and appropriate treatment may help reduce incidence of clinical manifestations and HCRU.

## Shareable abstract

**Patients with EGPA had varied manifestations, were treated with multiple therapies (including OCS), and had frequent healthcare visits, indicating a substantial disease burden. Earlier diagnosis and appropriate treatment may help reduce this burden.** https://bit.ly/46luUK3

## Introduction

Eosinophilic granulomatosis with polyangiitis (EGPA) is a rare systemic condition, typically characterised by vasculitis of small-to-medium vessels, elevated blood eosinophil count ≥1000 cells·μL^−1^ and extravascular eosinophilic inflammation, with the presence of asthma and/or nasal polyps [1, 2]. The manifestations of EGPA are heterogeneous, can affect multiple organ systems and can cause serious, or even life-threatening, organ damage if untreated [3, 4]. EGPA is classified among the antineutrophil cytoplasmic antibody (ANCA)-associated vasculitides (AAV) [1], and ∼30–40% of patients have detectable ANCA, which targets myeloperoxidase in the majority (>90%) of cases [5, 6]. Before developing EGPA, patients typically experience sequential respiratory tract and eosinophilic manifestations, then vasculitis, although phases often overlap and not all patients follow this progression [7]. Because of the complex histopathology of EGPA, the heterogeneous clinical presentation, and the prodromal and eosinophilic phases that can precede vasculitis development for months or years, a confirmed diagnosis of EGPA is challenging and often delayed [3, 4, 8, 9].

The treatment of EGPA relies heavily on oral corticosteroids (OCS), often in combination with immunosuppressive therapies or biologics, to first induce and then maintain remission as well as to treat relapses [10–12]. OCS and many immunosuppressive therapies are associated with substantial adverse effects [12–14]. Incidence of adverse effects increases with both OCS dose and duration of use, and can further contribute to the overall disease burden. Chronic OCS use may also be associated with greater long-term damage in vasculitides including EGPA [12, 15, 16].

Several biologics are under investigation for use in EGPA [16, 17]. Rituximab, an anti-CD20 monoclonal antibody that targets B-cells, is approved for the treatment of related AAVs, microscopic polyangiitis and granulomatosis with polyangiitis [18]. However, evidence that rituximab may also offer treatment benefit for patients with EGPA is limited to observational studies [19, 20]. Biologics such as the anti-interleukin (IL)-5 monoclonal antibodies mepolizumab and reslizumab, or those targeting the IL-5 receptor (benralizumab), are also potential treatments in EGPA, owing to their mechanism of action in depleting eosinophil levels [21]; each is approved for the treatment of severe eosinophilic asthma [22–24]. To date, only mepolizumab is approved for the treatment of EGPA, in multiple regions worldwide (European approval in 2021), in addition to other eosinophilic-driven diseases [24–26]. This approval followed the results of the Phase III MIRRA study, in which patients with relapsing or refractory EGPA treated with mepolizumab spent more time in remission, had reduced relapse rates and reduced OCS use compared with those receiving placebo [27, 28].

Estimations of the prevalence of EGPA vary from 2.0 to 30.4 cases per million individuals [29]. Owing to the rarity of the disease, studies assessing treatment patterns, disease manifestations, clinical outcomes and healthcare burden for patients with EGPA in Europe are limited. However, data from US studies demonstrate that healthcare resource utilisation (HCRU) by patients with EGPA is considerable [29, 30]. This study aimed to characterise patient demographic and clinical characteristics, diagnostic assessments, treatment patterns, clinical outcomes and HCRU for a large cohort of patients with EGPA in Europe.

## Methods

### Study design

This retrospective, non-interventional, longitudinal study (GSK ID: 214661) recruited cross-specialty physicians (rheumatologists, pulmonologists, allergists and immunologists) from five European countries (France, Germany, Italy, Spain and the UK) to conduct medical chart reviews of patients diagnosed with EGPA. ∼40 physicians from each country were planned to be recruited.

**FIGURE 1:**
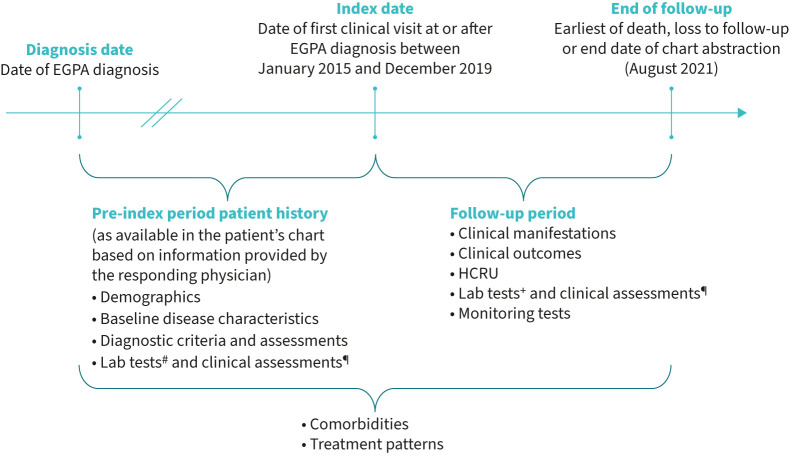
Study design. EGPA: eosinophilic granulomatosis with polyangiitis; HCRU: healthcare resource utilisation. ^#^: antineutrophil cytoplasmic antibodies (ANCA), blood eosinophil count, C-reactive protein, serum creatinine, five-factor score; ^¶^: Birmingham Vasculitis Activity Score, Paediatric Vasculitis Activity Score, Vasculitis Damage Index (VDI), Paediatric VDI; ^+^: ANCA, lung function.

The index date was defined as the date of the patient's first clinical visit with the physician between January 2015 and December 2019. The patient could be newly diagnosed with EGPA at index date or already diagnosed with EGPA. The end of follow-up was the earliest of death, loss to follow-up or the date of chart abstraction (figure 1). For patients diagnosed with EGPA before the index date, a patient history from EGPA diagnosis was collected using patient charts. Between the index date and the end of follow-up, a more detailed data set of patient information was collected based on patient charts, consisting of treatment patterns from EGPA diagnosis until end of follow-up, clinical manifestations, clinical outcomes and HCRU.

Physicians were blind to the identity of the study sponsor and the study sponsor was blind to the identities of the physicians and the patients.

### Data collection

Data were collected using a standardised electronic chart review form (eCRF) which ensured data integrity by incorporating quality control measures, including an automated consistency check and identification of physicians who had short response times or convenient response selection, who were then excluded from the analysis. A random temporal date shift was added to each patient to preserve data anonymity.

The eCRF included a 5-min screening survey which confirmed eligibility of the participating physician. Abstraction of data from medical charts and completion of the eCRF was anticipated to take ∼40 min per medical chart.

### Physician eligibility criteria

Physicians were identified from a directory of vetted physicians (Leaders in Medicine Atlas) and engaged by an external physician panel vendor (Medefield America Inc.). Specialists in rheumatology, allergy, pulmonology or immunology were invited to participate if they had access to the complete medical records of at least one eligible patient with EGPA and were the primary healthcare provider for those patients. Further details on the physician recruitment process are given in the supplementary methods.

### Patient eligibility criteria

Eligible patients had a physician-confirmed diagnosis of EGPA, were ≥12 years of age at the time of diagnosis and had at least 1 year of follow-up data available from the index date (except for patients who died within 1 year of index date).

To avoid bias in the selection of patient charts, physicians with more than one eligible chart selected charts based on a randomised sequence of letters. The interactive randomisation program produced a random letter and requested the physician to pull the first eligible patient chart with the last name that corresponded to the random letter. If no eligible patient had a last name beginning with the first letter in the sequence, the next letter in the sequence was shown.

### Outcomes and assessments

Physician characteristics were recorded on the date of the physician's chart abstractions and included country, specialty, practice setting, practice size, years in practice and mean number of unique patients with EGPA treated in the preceding 12 months.

Patient demographics, disease characteristics and diagnostic assessments were summarised between EGPA diagnosis and index date or at index date (unless otherwise stated), and comorbidities were summarised between EGPA diagnosis and the end of follow-up. Pre-index laboratory tests (ANCA, blood eosinophil count, C-reactive protein, serum creatinine, five-factor score) and clinical assessments (Birmingham Vasculitis Activity Score (BVAS), Paediatric Vasculitis Activity Score (PVAS), Vasculitis Damage Index (VDI), Paediatric Vasculitis Damage Index (PVDI)) were also assessed between EGPA diagnosis and index dates.

Measures of treatment patterns, including number and type of treatment, duration (OCS only) and maximum maintenance OCS dose where available, were collected for the period from EGPA diagnosis to the end of follow-up. Ongoing therapies for EGPA at the end of follow-up were also reported.

Details of clinical manifestations and their severity were collected from index date to the end of follow-up and were summarised by organ involvement. Clinical outcomes were collected for the same period and included achievement of remission and occurrence of relapse. Remission was defined as absence of disease activity (BVAS=0) and OCS dosage ≤4.0 mg·day^−1^, or other physician-defined remission. Relapse was defined as recurrence or worsening of EGPA symptoms requiring an increase in OCS dose, increase/change in dose of immunosuppressive therapy, hospitalisation or other physician-defined relapse. Outcome measures included cumulative duration of remission, time from diagnosis to first remission, number of relapses, real-world relapse-free survival (time from diagnosis to earliest relapse) and overall survival (time from diagnosis to death from any cause).

HCRU data, including EGPA-related outpatient visits, hospitalisations and emergency department (ED) visits, and tests related to the monitoring of clinical conditions, were collected from index date to the end of follow-up. Post-index laboratory tests (ANCA, lung function) and clinical assessments (BVAS, PVAS, VDI, PVDI) were reported between index date and the end of follow-up.

### Statistical analysis

Data for demographic and disease characteristics, comorbidities, diagnostic tests and treatment patterns were reported using descriptive statistics. Mean±sd and median (interquartile range (IQR)) values were used to summarise continuous variables; frequency distributions and proportions (n (%)) were used for categorical variables. Analyses of time to clinical manifestation (relapse-free survival and overall survival) were evaluated using the Kaplan–Meier (KM) methods and median times to event (if reached) were reported with 95% confidence intervals. Where median values were not reached, the restricted mean survival time (RMST) was calculated to estimate survival over a 6-year period. RMST measures the average event-free survival time as estimated by the area under the KM curve over a specified period of time and is unaffected by follow-up time or study-specific censoring distributions.

HCRU data were reported as mean±sd and median (IQR) number of events per patient per year and the proportion of patients with occurrence of the outcome of interest.

### Ethics

This study complied with all applicable laws regarding subject privacy including European General Data Protection Regulation requirements. No direct patient contact or primary collection of individual patient data occurred. Study results were from aggregate analyses that omitted patient identification; therefore, informed consent was not required.

## Results

### Physician characteristics

Medical charts were reviewed by 204 physicians, comprising 38–45 physicians from each country. Physicians specialised in rheumatology (89; 43.6%), pulmonology (76; 37.3%), allergy (26; 12.7%) or immunology (13; 6.4%), and most indicated that their primary practice setting was academic (134; 65.7%). Most physicians had been in practice 11–20 years (95; 46.6%) and a further 78 (38.2%) had been in practice longer than 20 years. The median (IQR) number of patients with EGPA treated per physician in the 12 months before chart abstraction was 10.0 (4.0–23.0) and median (IQR) number of charts completed per physician was 2.0 (1.0–2.0) (supplementary table S1).

### Patient demographics, disease characteristics and comorbidities

**FIGURE 2:**
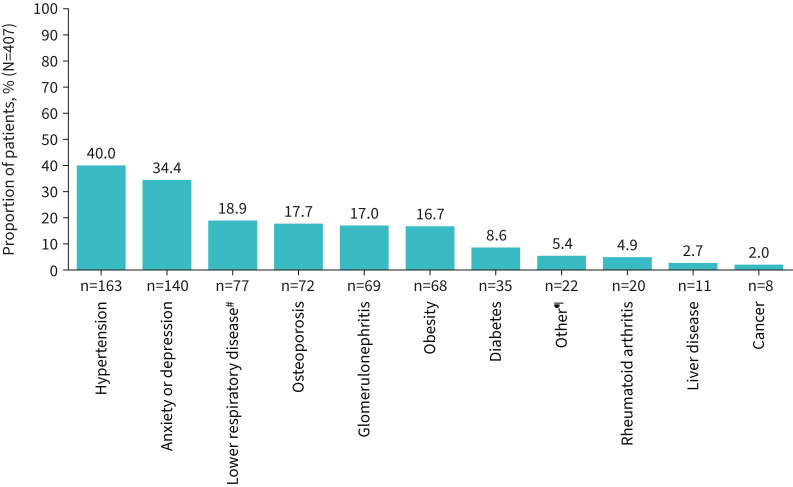
Comorbidities of patients with eosinophilic granulomatosis with polyangiitis (EGPA), between EGPA diagnosis and the end of follow-up (N=407). ^#^: other than asthma/COPD; ^¶^: “other” comorbidities (submitted as free-text): hay fever (n=4), sinusitis (n=3), osteoporosis (n=2), atrial fibrillation, bowel infarction, cardiac ischaemia, coronary artery disease, fatty liver disease, heart failure, hives, hypothyroidism, multiple sclerosis, obstructive sleep apnoea symptoms, oligoarthritis, peripheral neuropathy, psoriasis, skin ulcer, strabismus (each n=1).

Medical chart data for 407 patients were collected: 80–85 patients per country (table 1). The mean±sd age at diagnosis was 43.2±14.9 years, and 231 (56.8%) patients were male. Few patients (24; 5.9%) were younger than 18 years of age at diagnosis. The median (IQR) duration of follow-up from index date was 2.2 (1.7–3.5) years, and the median (IQR) disease duration from diagnosis to end of follow-up was 2.5 (1.8–4.1) years. Most patients (359; 88.2%) were diagnosed after 2015 and 162 (39.8%) patients were diagnosed on their index date. EGPA phase (physician reported) was most reported as eosinophilic (220 patients; 54.1%) or vasculitic (125; 30.7%). Vasculitis as a comorbidity was specifically reported for 197 (48.4%) patients. The most common comorbidities were hypertension (163 patients; 40.0%) and anxiety or depression (140; 34.4%) (figure 2). A large proportion of patients were also reported to have asthma (299; 73.5%), a common disease feature associated with EGPA.

**TABLE 1:** Patient demographics, baseline disease characteristics and diagnostic assessments (N=407)

**Patient demographics**	
Country	
France	81 (19.9)
Germany	80 (19.7)
Italy	80 (19.7)
Spain	85 (20.9)
UK	81 (19.9)
Age at EGPA diagnosis, years, mean±sd	43.2±14.9
Paediatric (age 12–17 years)	24 (5.9)
Adult (≥18 years)	383 (94.1)
Age on index date, years, mean±sd	44.1±15.0
Male	231 (56.8)
Smoking status^#^	
Never smoked	193 (47.4)
Former smoker	155 (38.1)
Current smoker	50 (12.3)
Unknown	9 (2.2)
Year of diagnosis	
Pre-2014 (before the study)	48 (11.8)
2015–2019 (during the study)	359 (88.2)
Duration between diagnosis and index date, years, median (IQR)	0.0 (0.0–0.3)
Patients with diagnosis on index date	162 (39.8)
**Patient disease and diagnostic characteristics**	
Diagnostic/classification criteria^¶^	
American College of Rheumatology	273 (67.1)
Chapel Hill Consensus Conference	109 (26.8)
Other^+^	20 (4.9)
Unknown	41 (10.1)
Diagnostic assessments^¶^	
Blood eosinophilia	381 (93.6)
Blood tests to screen for autoimmunity (*e.g.* ANCA)	372 (91.4)
Imaging scans of affected organs	338 (83.0)
Biopsy to detect extravascular eosinophils	203 (49.9)
Test to detect neuropathy	157 (38.6)
Other test of affected organs^§^	19 (4.7)
Unknown	3 (0.7)
Number of diagnostic assessments^*f*^	
1–3	98 (24.1)
4–6	189 (46.4)
7–12	117 (28.7)
Physician-reported phase of EGPA^##^	
Eosinophilic	220 (54.1)
Vasculitic	125 (30.7)
Prodromal	36 (8.8)
Unknown	26 (6.4)
Patients with asthma	299 (73.5)
Asthma diagnosis date before EGPA diagnosis^¶¶^	160 (53.5)
Time from asthma diagnosis to EGPA diagnosis, years	
Median (IQR)	1.8 (0.2–5.6)
Mean±sd	4.3±6.5
Asthma diagnosis date after EGPA diagnosis^¶¶^	45 (15.1)
Asthma with no diagnosis date^¶¶^	94 (31.4)

Data are presented as n (%) unless otherwise stated. EGPA: eosinophilic granulomatosis with polyangiitis; IQR: interquartile range; ANCA: antineutrophil cytoplasmic antibodies. ^#^: smoking status was assessed at chart abstraction. ^¶^: diagnostic criteria and diagnostic assessments were not mutually exclusive categories. ^+^: other diagnostic criteria included European League Against Rheumatism (n=8 patients), British Thoracic Society (n=5), Société de Pneumologie de Langue Française (n=2), posterior reversible encephalopathy syndrome (n=1); five charts also indicated diagnostic criteria guided by other clinical assessments (*e.g.* British Society of Rheumatology, blood tests, biopsies) and local guidelines (n=5). ^§^: other tests not included in the chart review form were entered as free text by the physician and included electromyography (n=2 patients), renal biopsy (n=2), rhinoscopy (n=2), bronchopulmonary biopsy (n=1), bronchoscopy (n=1), capillary microscopy (n=1), cardiology (n=1), endoscopy (n=1), and other tests associated with eye, kidney, skin, lung and urinary systems (n=7). ^*f*^: all the categories of diagnostic assessment were counted for this statistic; for example, if a physician indicated that a patient had an imaging scan of affected organs, and then further specified that the patient had an abdominal computed tomography scan and a chest radiograph, this would count as two diagnostic assessments. ^##^: assessed on or before index date. ^¶¶^: percentage based on 299 patients with asthma.

### Diagnostic assessments

Patients underwent multiple diagnostic assessments before or at index date, most commonly blood eosinophilia tests (381 patients; 93.6%), blood tests to screen for autoimmunity (372; 91.4%), imaging scans of affected organs (338; 83.0%) and biopsy to detect extravascular eosinophils (203; 49.9%) (table 1). The median (IQR) most recent pre-index blood eosinophil count was 1500 (600–3300) cells·μL^−1^. ANCA testing was reported for 319 patients (78.4%) pre-index, and 328 (80.6%) pre- or post-index. Further details of other pre-index date laboratory tests and assessments are detailed in supplementary table S2; American College of Rheumatology classification criteria were most commonly used (273 cases; 67.1%), followed by the Chapel Hill Consensus Conference criteria (109 cases; 26.8%) (table 1) [2, 31].

### Treatment patterns

Most patients received multiple types of therapy between diagnosis and the end of follow-up; 305 (74.9%) patients were treated with three or more different therapies (table 2). OCS use was nearly ubiquitous, used by 402 (98.8%) patients. Across all OCS drugs, the median (IQR) cumulative duration of OCS use was 22.2 (11.5–34.8) months with a median (IQR) maximum daily dose of 30.0 (10.0–50.0) mg (prednisone-equivalent). Most patients were also treated with immunosuppressive or other therapies (260; 63.9%), mainly azathioprine (110; 27.0%), cyclophosphamide (78; 19.2%) and methotrexate (77; 18.9%). In addition, 185 (45.5%) patients received a biologic therapy (during the follow-up period all biologics use for the treatment of EGPA was off-label), which was initiated a median (IQR) of 1.4 (0.5–2.7) years after diagnosis. The most used biologics were mepolizumab and rituximab, each used by 74 (18.2%) patients. As well as taking OCS, immunosuppressive therapies or biologics, 302 (74.2%) patients received other treatments for EGPA-related manifestations and 238 (58.5%) patients received therapies for the management of other clinical conditions. At the end of follow-up, 254 (62.4%) patients were receiving ongoing OCS, 183 (45.0%) were receiving immunosuppressive or other therapies for EGPA, most commonly azathioprine (17.4%) and methotrexate (11.8%), and 130 (31.9%) were receiving biologics, most commonly mepolizumab (13.5%) and rituximab (9.6%).

**TABLE 2:** Treatment patterns, assessed between EGPA diagnosis and the end of follow-up (N=407)

**Time from diagnosis to initiation of therapy, years, median (IQR)**	0.0 (0.0–0.1)
**Number of distinct EGPA treatment types used** **^#^**	
Mean±sd	3.9±1.8
By category	
1–2	102 (25.1)
3–4	166 (40.8)
5–7	126 (31.0)
≥8	13 (3.2)
**EGPA therapies by treatment category**	
OCS	402 (98.8)
Prednisone or prednisolone	349 (85.7)
Methylprednisolone	98 (24.1)
Cortisone	19 (4.7)
Duration across all OCS, months, median (IQR)^¶^	22.2 (11.5–34.8)
Maximum daily maintenance dose for OCS, mg, median (IQR)^+^	30.0 (10.0–50.0)
** **Immunosuppressive therapies and other therapies for EGPA (used by >5% of patients)^§^	260 (63.9)
Azathioprine	110 (27.0)
Cyclophosphamide	78 (19.2)
Methotrexate	77 (18.9)
Mycophenolate	32 (7.9)
Biologics	185 (45.5)
Mepolizumab	74 (18.2)
Rituximab	74 (18.2)
Benralizumab	26 (6.4)
Omalizumab	18 (4.4)
Reslizumab	16 (3.9)
Dupilumab	1 (0.2)
Time from diagnosis to initiation of biologic therapy, years, median (IQR)	1.4 (0.5–2.7)
Other treatments for EGPA-related clinical manifestations (used by >5% of patients)^*f*^	302 (74.2)
Budesonide–formoterol	160 (39.3)
Albuterol	119 (29.2)
Analgesics	96 (23.6)
Montelukast	62 (15.2)
Ramipril	55 (13.5)
Tiotropium	45 (11.1)
Ipratropium bromide	40 (9.8)
Valsartan	27 (6.6)
**Treatments related to the management of other clinical conditions** **^##^**	238 (58.5)
**Ongoing EGPA therapies at the end of follow-up**	
OCS	254 (62.4)
** **Immunosuppressive therapies and other therapies for EGPA (used by >5% of patients)	183 (45.0)
Azathioprine	71 (17.4)
Methotrexate	48 (11.8)
Mycophenolate	25 (6.1)
** **Biologics (used by >5% of patients)	130 (31.9)
Mepolizumab	55 (13.5)
Rituximab	39 (9.6)
Benralizumab	22 (5.4)
** **Other treatments for EGPA-related clinical manifestations	271 (66.6)

Data are presented as n (%) unless otherwise stated. EGPA: eosinophilic granulomatosis with polyangiitis; IQR: interquartile range; OCS: oral corticosteroid. **^#^**: receipt of one or multiple OCS drugs was counted as a single therapy. ^¶^: for patients who received more than one prescription for a drug, the duration of therapy was calculated as the sum of durations of therapy across all prescriptions. ^+^: among 314 patients for whom maximum daily dose (prednisone-equivalent dose) was reported; data for patients with a reported maximum daily dose >60 mg were removed as these values were considered to reflect burst treatment episodes; for patients who received more than one prescription for a drug, the maximum dosage was reported as the maximum dosage across all prescriptions. ^§^: other immunosuppressive and other therapies included cyclosporine (n=15 patients), hydroxyurea (n=2), immunoglobulin (intravenous) (n=9), interferon-α (n=4), leflunomide (n=8) and plasma exchange (n=7). ^*f*^: other therapies for EGPA-related clinical manifestations included doxazosin (n=7), levalbuterol (n=5), theophylline (n=8), zafirlukast (n=2). ^##^: treatment patterns for management of other clinical conditions were assessed between the index date and end of follow-up, and included treatments for low bone mineral density, infections related to EGPA therapies, recovering cytopenia, haemorrhagic cystitis, hepatotoxicity and gastrointestinal toxicity; and thyroid hormone replacement therapy, insulin and folic acid supplements.

### Clinical manifestations and clinical outcomes

During the follow-up period, patients experienced a mean±sd of 4.1±4.0 distinct EGPA clinical manifestations and 108 (26.5%) had ≥6 distinct manifestations (table 3). The organs most involved were the lungs (227 patients; 55.8%), ear, nose and throat (217; 53.3%), and skin (170; 41.8%). The most common lung manifestations were shortness of breath (151 patients; 37.1%), lung infiltrates (105; 25.8%) and severe asthma (101; 24.8%). Constitutional manifestations (fatigue and myalgia/arthralgia) affected 198 (48.6%) patients. Manifestations generally considered vasculitic in origin (rather than resulting from eosinophilia) were less common and included renal manifestations, present in 78 (19.2%) patients (specifically glomerulonephritis: 43 (10.6%); proteinuria: 39 (9.6%); haematuria: 22 (5.4%)), and purpura in 65 (16.0%) patients [32]. 42 (10.3%) patients had biopsy-confirmed eosinophilic vasculitis or eosinophilic inflammation during follow-up. Gastrointestinal manifestations affected 79 (19.4%) patients, 45 (11.1%) had cardiovascular manifestations (most commonly cardiac arrhythmia: 18 (4.4%); least commonly heart failure: 7 (1.7%)), and 75 (18.4%) had neuropsychiatric manifestations (most commonly peripheral neuropathy: 51 (12.5%); least commonly psychosis and stroke: 5 (1.2%) each).

**TABLE 3:** Clinical manifestations and severity ratings for patients with EGPA, assessed between the index date and end of follow-up

	Patients with clinical manifestation^#^	Proportion of manifestations classified as mild^¶^	Proportion of manifestations classified as moderate^¶^	Proportion of manifestations classified as severe^¶^
**Number of distinct manifestations, mean±sd**	4.1±4.0	NA	NA	NA
**Number of distinct manifestations by category**		NA	NA	NA
0	62 (15.2)	NA	NA	NA
1–2	104 (25.6)	NA	NA	NA
3–5	133 (32.7)	NA	NA	NA
6–8	61 (15.0)	NA	NA	NA
9–12	28 (6.9)	NA	NA	NA
≥13	19 (4.7)	NA	NA	NA
**Clinical manifestations by organ involvement**				
Lung	**227 (55.8)**			
Shortness of breath	151 (37.1)	31 (20.5)	88 (58.3)	29 (19.2)
Lung infiltrates	105 (25.8)	30 (28.6)	56 (53.3)	18 (17.1)
Severe asthma	101 (24.8)	9 (8.9)	37 (36.6)	54 (53.5)
Pleural effusion	21 (5.2)	5 (23.8)	12 (57.1)	4 (19.0)
Alveolar haemorrhage	15 (3.7)	6 (40.0)	3 (20.0)	6 (40.0)
** **Ear, nose and throat	**217 (53.3)**			
Allergic rhinitis	140 (34.4)	32 (22.9)	89 (63.6)	13 (9.3)
Paranasal sinusitis	90 (22.1)	25 (27.8)	51 (56.7)	12 (13.3)
Nasal polyposis	89 (21.9)	21 (23.6)	53 (59.6)	13 (14.6)
Otitis media	14 (3.4)	6 (42.9)	6 (42.9)	2 (14.3)
Constitutional	**198 (48.6)**			
Fatigue	174 (42.8)	41 (23.6)	107 (61.5)	25 (14.4)
Myalgia/arthralgia	111 (27.3)	35 (31.5)	68 (61.3)	8 (7.2)
Skin	**170 (41.8)**			
Itch	78 (19.2)	27 (34.6)	43 (55.1)	8 (10.3)
Urticaria	74 (18.2)	22 (29.7)	43 (58.1)	7 (9.5)
Purpura	65 (16.0)	18 (27.7)	39 (60.0)	7 (10.8)
Ulcers	27 (6.6%)	3 (11.1)	19 (70.4)	4 (14.8)
Gastrointestinal	**79 (19.4)**			
Abdominal pain	43 (10.6)	17 (39.5)	24 (55.8)	2 (4.7)
Diarrhoea	31 (7.6)	16 (51.6)	10 (32.3)	4 (12.9)
Nausea/vomiting	25 (6.1)	11 (44.0)	12 (48.0)	1 (4.0)
Gastrointestinal bleeding	17 (4.2)	5 (29.4)	9 (52.9)	1 (5.9)
Renal	**78 (19.2)**			
Glomerulonephritis	43 (10.6)	15 (34.9)	23 (53.5)	3 (7.0)
Proteinuria	39 (9.6)	18 (46.2)	18 (46.2)	2 (5.1)
Haematuria	22 (5.4)	7 (31.8)	14 (63.6)	0
Cardiovascular	**45 (11.1)**			
Cardiac arrhythmia	18 (4.4)	7 (38.9)	7 (38.9)	2 (11.1)
Ischaemic heart disease	14 (3.4)	3 (21.4)	9 (64.3)	1 (7.1)
Cardiomyopathy	9 (2.2)	3 (33.3)	4 (44.4)	1 (11.1)
Peripheral vascular disease	9 (2.2)	3 (33.3)	3 (33.3)	1 (11.1)
Valvular disease	9 (2.2)	3 (33.3)	3 (33.3)	1 (11.1)
Pericarditis	8 (2.0)	1 (12.5)	5 (62.5)	1 (12.5)
Heart failure^+^	7 (1.7)	NA	NA	NA
* * Functional capacity class				
I	0			
II	4 (57.1)			
III	2 (28.6)			
IV	0			
Objective assessment				
A	1 (14.3)			
B	3 (42.9)			
C	2 (28.6)			
D	0			
Neuropsychiatric	**75 (18.4)**			
Peripheral neuropathy	51 (12.5)	16 (31.4)	24 (47.1)	9 (17.6)
Mononeuritis	22 (5.4)	5 (22.7)	12 (54.5)	5 (22.7)
Cranial nerve palsies or involvement	6 (1.5)	4 (66.7)	2 (33.3)	0
Psychosis	5 (1.2)	3 (60.0)	0	0
Stroke	5 (1.2)	3 (60.0)	1 (20.0)	1 (20.0)
Biopsy-confirmed eosinophilic vasculitis or eosinophilic inflammation	**42 (10.3)**	10 (23.8)	26 (61.9)	5 (11.9)

Data are presented as n (%) unless otherwise stated. EGPA: eosinophilic granulomatosis with polyangiitis; NA: not available. Bold type indicates proportion of patients with manifestation at the organ level. ^#^: N=407; ^¶^: severity was documented at first occurrence on or after index date and was classified as mild=present but minimal impact, moderate=significant impact on daily activities, severe=incapacitating, or unknown; ^+^: severity of heart failure was defined by functional capacity (class I to IV; where I is no limitation on physical activity and IV is symptoms of heart failure at rest and increasing discomfort with physical activity) and objective assessment (class A to D; where A is no evidence of cardiovascular disease and D is severe cardiovascular disease) based on New York Heart Association heart failure criteria [46].

For most conditions the majority of manifestations were considered moderate (significant impact on daily activities) or severe (incapacitating) (table 3).

During the follow-up period, 242 (59.5%) patients experienced remission (table 4), with a median (IQR) time of 14.5 (7.5–26.9) months from diagnosis to first remission, and 78 (19.2%) experienced at least one relapse. For 69 (17.0%) patients relapse occurred within 6 years of diagnosis and the estimated relapse-free RMST by year 6 was 5.03 years. 11 patients died within 6 years of diagnosis, and the overall RMST by year 6 was 5.84 years.

**TABLE 4:** Clinical outcomes for patients with EGPA, assessed between the index date and end of follow-up (N=407)

**Patients who experienced remission** **^#^**	242 (59.5)
** **Cumulative duration of time spent in remission(s), months, median (IQR)	12.0 (5.2–22.2)
** **Duration of individual episodes of remission, months, median (IQR)	11.6 (4.9–20.8)
** **Time from diagnosis to first remission, months, median (IQR)	14.5 (7.5–26.9)
**Patients who experienced a relapse** **^¶^**	78 (19.2)
** **Number of relapses (among patients with a relapse), PPPY, median (IQR)	0.6 (0.3–1.0)
**Estimated relapse-free survival time, RMST, years**	5.03
** **Patients who experienced a relapse within 6-year RMST period from diagnosis	69 (17.0)
** **Patients who died within 6-year RMST period from diagnosis	11 (2.7)

Data are presented as n (%) unless otherwise stated. EGPA: eosinophilic granulomatosis with polyangiitis; IQR: interquartile range; PPPY: per person per year; RMST: restricted mean survival time. ^#^: remission was defined by the physician; definitions used to classify remission were not mutually exclusive and included Birmingham Vasculitis Activity Score=0 (24 physicians; 16.4%), oral corticosteroid dosage ≤4 mg·day^−1^ (99 physicians; 67.8%) and other (17 physicians; 11.6%); missing/not reported for 19 physicians (13.0%). ^¶^: relapse was defined as recurrence or worsening of EGPA symptoms requiring an increase in oral corticosteroid dose, an increase/change in dose of immunosuppressive therapy, hospitalisation or other physician-defined relapse.

### EGPA-related HCRU

**FIGURE 3:**
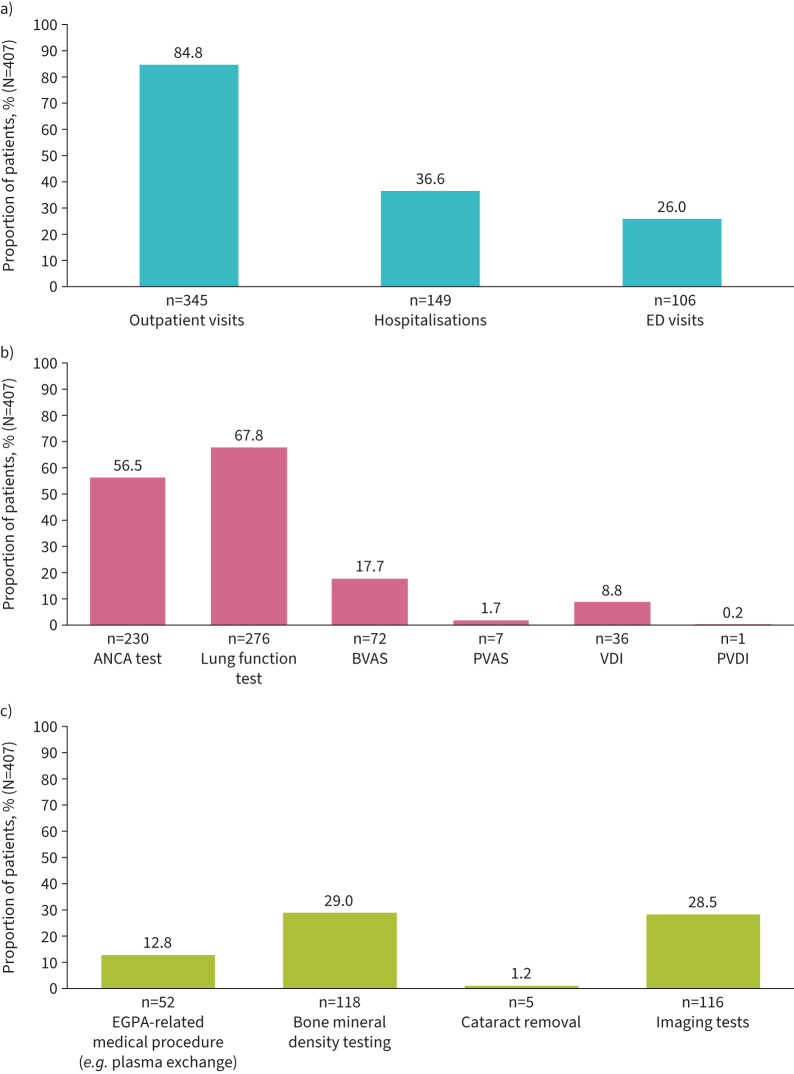
Healthcare resource utilisation (N=407): a) Eosinophilic granulomatosis with polyangiitis (EGPA)-related healthcare visits (between EGPA diagnosis and the end of follow-up); b) Lab tests and assessments (between index and the end of follow-up); c) Tests related to the monitoring of clinical conditions (between index and the end of follow-up). ED: emergency department; ANCA: antineutrophil cytoplasmic antibodies; BVAS: Birmingham Vasculitis Activity Score; PVAS: Paediatric Vasculitis Activity Score; VDI: Vasculitis Damage Index; PVDI: Paediatric VDI.

During follow-up, 106 (26.0%) patients visited the ED, 149 (36.6%) were hospitalised and 345 (84.8%) made outpatient visits (figure 3a) for EGPA-related reasons. The mean±sd number of visits per patient per year was 0.3±1.0 for EGPA-related ED visits, 0.5±1.3 for hospitalisations and 5.9±38.0 for outpatient visits. For the 149 patients who were hospitalised, the mean±sd duration of stay was 8.4±6.6 days. The proportions of patients having post-index laboratory tests and assessments are shown in figure 3b; lung function tests were the most frequently conducted assessment (276; 67.8%). Approximately one-third of patients had bone mineral density testing (29.0%) or imaging tests (28.5%) for the monitoring of clinical conditions during follow-up (figure 3c).

## Discussion

To our knowledge, this real-world, retrospective medical chart review study is the largest multi-country assessment of EGPA manifestations, treatment patterns and HCRU in Europe, representing further evidence of the substantial burden associated with this rare disease.

Patients with EGPA included in this study were mostly adults with paediatric patients representing ∼6% of cases. Patients younger than 12 years of age were excluded and paediatricians were not recruited, which may have limited the number of paediatric cases identified. Before receiving a diagnosis, patients underwent multiple diagnostic assessments, and physicians differed in their use of classification criteria. This confirms the inherent challenges associated with diagnosing this complex disease and may reflect geographical differences in diagnostic pathways. Notably, >90% of patients underwent blood tests for autoimmunity as part of their diagnostic assessments, and 78% had an ANCA test pre-index. Accounting for the importance of ANCA status to the diagnostic workup for suspected EGPA, ANCA testing would be expected to be closer to 100% [33]. Half of patients had biopsy for extravascular eosinophils and clinical assessments such as BVAS and VDI were relatively uncommon, reported for 25% and 13% of patients pre-index, respectively.

In an earlier retrospective, long-term follow-up study of patients with EGPA in the French Vasculitis Study Group cohort, the mean duration from asthma diagnosis to EGPA diagnosis was >9 years [3]. A third of patients were diagnosed before 1996 and 96% of these had comorbid asthma compared with 88% of patients diagnosed after 1996. This pattern, whereby symptoms of adult onset asthma develop before other symptoms of EGPA (*i.e.* prodromal phase), is well established [7]. In the current study 88% of patients were diagnosed with EGPA between 2015 and 2019, and 74% had asthma diagnoses. Although in the majority of cases (at least 54%) asthma preceded EGPA, this is lower than the earlier study, and there was a shorter mean duration from asthma diagnosis to EGPA diagnosis (4.3 years). This difference is, in part, likely due to the increased awareness, changes in classification criteria and improved diagnostic tools for EGPA over time [10], and highlights the importance of recognising EGPA symptoms in patients diagnosed with asthma. Additional comorbid or associated conditions described at the time of diagnosis included hypertension, anxiety and depression, and other lower respiratory disease, typifying the complexity of presenting symptoms.

Most patients experienced three or more distinct manifestations (mean±sd: 4.1±4.0) during the follow-up period, a large proportion of which were classified as moderate or severe at the first occurrence. This is lower than reported in a US cohort study for which the mean±sd number of manifestations through to the end of the study was 6.6±2.3, over a longer mean duration of follow-up of 7 years. The nature of the clinical manifestations was similar between the two studies with lung, ear/nose/throat and constitutional symptoms being the most frequently reported manifestations [4]. Similarly, vasculitic manifestations such as renal manifestations and purpura (19% and 16% in the current study, respectively) were moderately common, in keeping with other reports [4]. Outcomes and treatment patterns in groups of patients with vasculitic manifestations were not assessed in the current study, but would be of interest in future investigations. Of note, in the current study almost 60% of patients achieved remission with a median time from diagnosis to first remission of longer than a year (14.5 months) and almost one in five patients experienced a relapse.

The results presented here highlight the continued reliance on OCS by patients with EGPA, which in turn has an associated burden in terms of OCS-related complications (*e.g.* osteoporosis, diabetes and infections) [4]. The high rate of relapse, despite OCS use, indicates that patients are not optimally controlled with current therapies and that treatment optimisation strategies are needed [34]. For most of the study period, there were no approved biologics for EGPA, and their use was only supported by low-grade evidence [35]. However, during the study period, mepolizumab was approved for eosinophilic asthma (2015), and the results of the Phase III MIRRA trial for mepolizumab in EGPA were available (2017) [28]. This led to multidisciplinary discussions and off-label use of biologics ahead of formal approval. Since the end of the study observation period (2021), mepolizumab has become the only biologic currently approved for EGPA [24–26], and increasing evidence from both clinical and real-world studies has emerged in support of the use of biologics targeting IL-5 signalling (*e.g.* mepolizumab, benralizumab and reslizumab) to control disease and reduce OCS use [10–12, 17, 28, 36–44]. The first results of the Phase III MANDARA clinical study have been recently presented, showing the non-inferiority of benralizumab *versus* mepolizumab in patients with relapsing/refractory EGPA [42]. In addition, a recent observational study of 49 patients with relapsing/refractory EGPA reported that mepolizumab and benralizumab improved respiratory outcomes, reduced blood eosinophil counts, showed OCS-sparing benefits and prompted the discontinuation of disease-modifying antirheumatic drugs [43]. Another retrospective study in patients with both severe eosinophilic asthma and EGPA treated for 12 months with mepolizumab or benralizumab showed EGPA remission rates of 45.4% (BVAS=0 and OCS dose ≤4 mg) or 68.2% (BVAS=0 and OCS dose ≤7.5 mg), depending on the criteria chosen [44]. In the current study, we found that only 45.5% of patients received a biologic therapy, with mepolizumab and rituximab being the most used biologics (18.2% each), suggesting that more patients with EGPA could benefit from biologics.

The HCRU identified in the present study aligns with recent studies (2021) of EGPA burden in the USA. A recent retrospective database study of patients with EGPA in the USA found that patients with EGPA incurred significantly greater all-cause healthcare costs, with more HCRU and OCS use than patients with asthma [34]. A second US database study found that 75% of patients with EGPA were prescribed OCS and over a third required hospitalisation [30]. Despite a high reliance on OCS, in the present study less than a third of patients had monitoring tests for bone health. Together these studies confirm the considerable HCRU burden conferred by EGPA, illustrating the importance of earlier diagnosis and treatment with newer therapies such as biologics to alleviate the HCRU burden and limit OCS use among patients.

This study was a retrospective analysis of patient medical charts and several limitations should be considered. Because of physician anonymity, an audit of medical records could not be performed to verify the accuracy of data abstracted from charts; however, quality control and validation processes were incorporated in the study design to maximise data integrity. The potential for reporting bias or selection bias cannot be excluded, and missing data or gaps in data collection may be non-random. However, we included a robust randomisation scheme for patient chart selection to mitigate potential selection biases. Details of EGPA manifestations were collected from the index date to the end of follow-up, so for patients who were diagnosed before the index date some manifestations after diagnosis may not have been recorded and estimates of time from diagnosis to first relapse may represent overestimates. In addition, future analyses should investigate the country-by-country variation in the diagnostic pathways and treatment approach to EGPA [45].

These real-world data demonstrate that EGPA is a serious and multi-systemic disease that presents a substantial burden on both patients and the healthcare system. Improvements in diagnostic and treatment approaches are needed to better manage and treat patients with EGPA.

## Data Availability

Data used for this publication were generated by Analysis Group. For access to anonymised subject level data, please contact Analysis Group.
